# All galls are divided into three or more parts: recursive enumeration of labeled histories for galled trees

**DOI:** 10.1186/s13015-023-00224-4

**Published:** 2023-02-13

**Authors:** Shaili Mathur, Noah A. Rosenberg

**Affiliations:** Department of Biology, Stanford University, Stanford, 94305 CA USA

**Keywords:** Galled trees, Labeled histories, Mathematical phylogenetics, Phylogenetic networks

## Abstract

**Objective:**

In mathematical phylogenetics, a labeled rooted binary tree topology can possess any of a number of labeled histories, each of which represents a possible temporal ordering of its coalescences. Labeled histories appear frequently in calculations that describe the combinatorics of phylogenetic trees. Here, we generalize the concept of labeled histories from rooted phylogenetic trees to rooted phylogenetic networks, specifically for the class of rooted phylogenetic networks known as rooted *galled trees*.

**Results:**

Extending a recursive algorithm for enumerating the labeled histories of a labeled tree topology, we present a method to enumerate the labeled histories associated with a labeled rooted galled tree. The method relies on a recursive decomposition by which each gall in a galled tree possesses three or more descendant subtrees. We exhaustively provide the numbers of labeled histories for all small galled trees, finding that each gall reduces the number of labeled histories relative to a specified galled tree that does not contain it.

**Conclusion:**

The results expand the set of structures for which labeled histories can be enumerated, extending a well-known calculation for phylogenetic trees to a class of phylogenetic networks.

## Introduction

Labeled histories represent a fundamental concept in mathematical phylogenetics, tabulating sequences in which the branching events that have given rise to a set of labeled lineages could have taken place. Given a set of labeled lineages at the leaves of a rooted binary tree, many topological relationships are possible for those lineages, each describing a *labeled topology*, each of which in turn is compatible with one or more *labeled histories* (p. 47 of [24]).

Labeled histories, sometimes also termed *ordered trees* [23] or *coalescence sequences* [20], have appeared in many types of studies. They arise in basic phylogenetic combinatorics, in which classes of phylogenetic trees are enumerated and their features assessed [11, 24]. In coalescent theory, which studies genetic lineages sampled in a population, the labeled histories, viewed backward in time from the present, describe the set of possible sequences in which the sampled gene lineages coalesce to a common ancestor [26]. Probability computations in coalescent theory often consider a set of labeled histories that is compatible with a desired tree shape [16, 17, 19]. Labeled histories arise frequently in the combinatorics of gene trees and species trees, in which labeled topologies for gene lineages sampled from a set of species are considered in relation to labeled topologies for the species themselves [5]. Algorithms that traverse tree spaces in searching for labeled topologies that could underlie molecular data also make use of labeled histories [13].

Fundamental results on labeled histories include the number of labeled histories possible for *n* labeled lineages [6] and the number of labeled histories for a specified labeled topology [3, 24] (see also problem 20 on p. 67 of [15]). The labeled topologies that, for a specified number of lineages, possess the largest number of labeled histories are also known [10].

Recently, much attention in mathematical phylogenetics has considered phylogenetic networks, generalizations of phylogenetic trees in which evolution has not necessarily proceeded in a tree-like manner [12]. Because biological phenomena such as admixture, horizontal gene transfer, hybridization, and the genetic exchanges that occur via migration can induce non-tree-like evolution for a set of biological groups, phylogenetic networks are increasingly relevant to a variety of biological problems.

Similar concepts to labeled histories can be defined for networks, in particular, those networks that are meant to represent evolution in time. Indeed, Bienvenu et al. [1] suggest the study of labeled histories for phylogenetic networks, focusing on tree–child networks. They pose the problem of enumerating the analogue of labeled histories for networks: the problem of enumerating labeled phylogenetic networks whose internal nodes are placed in distinct temporal orders, or rankings, but that share an unranked labeled structure in common (p. 656). Here, we solve this enumeration for a class of phylogenetic networks, namely the rooted labeled *galled trees*. Galled trees, which first emerged from the study of ancestral recombination graphs [9, 22], represent a relatively simple type of network structure, a subset of the tree–child networks.

We first introduce precise notions of galled trees and labeled histories. Next, we perform the enumeration of labeled histories for an example labeled galled tree. The example is followed by the general computation of the number of labeled histories for an arbitrary labeled galled tree. We then use the general computation to exhaustively count labeled histories for all labeled galled trees with at most 6 leaves. We conclude with a discussion.

## Preliminaries

### Definitions

Our focus is on rooted galled trees, a type of rooted binary phylogenetic network. Following Definition 1.1 of Bienvenu et al. [1], we consider a *rooted binary phylogenetic network* to be a directed acyclic graph such that (i) there is a unique *root node* with in-degree 0 and out-degree 2, (ii) all *leaf nodes* have in-degree 1 and out-degree 0, (iii) non-leaf and non-root nodes have either in-degree 2 and out-degree 1 or in-degree 1 and out-degree 2, and (iv) all edges are directed away from the root. Nodes with in-degree 2 and out-degree 1 are termed *reticulation nodes*, and nodes with in-degree 1 and out-degree 2 are *tree nodes*.

**Fig. 1:**
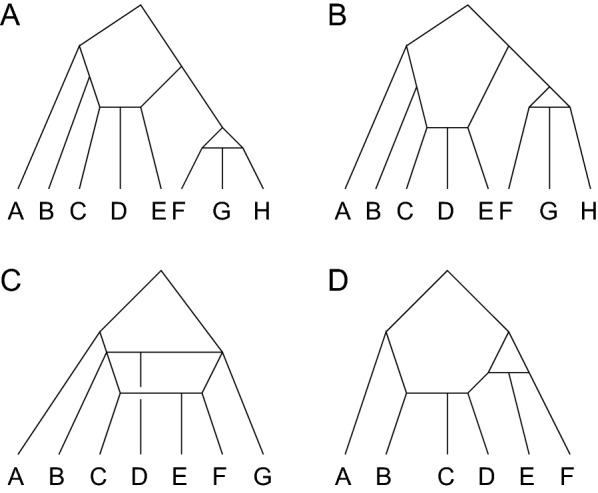
Galled trees. **A** A galled tree with two galls. **B** A galled tree with the same labeled topology as **A** but with a different labeled history. **C** A network that is not a galled tree because it contains nested cycles. **D** A network that is not a galled tree because it has cycles that share vertices. This network would be included in the class of galled networks [8], which is distinct from the class of galled trees

Note that although, as a directed acyclic graph, a rooted binary phylogenetic network has no directed cycles, if the sense of direction is removed, then the associated undirected graph can possess cycles. If this undirected graph has no cycles, then the network is simply a tree. The undirected graph of a galled tree does not contain nested cycles (Fig. 1).

A rooted *galled tree* is a rooted binary phylogenetic network in which (i) each reticulation node $a_r$ has a unique ancestor node *r* such that exactly two nonoverlapping paths of edges exist from *r* to $a_r$; if the direction of edges is ignored, then *r*, $a_r$, and these two paths form a cycle $C_r$, known as a *gall*. In addition, (ii) for reticulation nodes $a_r$ and $a_s$, the sets of nodes in the galls $C_r$ associated with $a_r$ and $C_s$ associated with $a_s$ are disjoint.

**Fig. 2:**
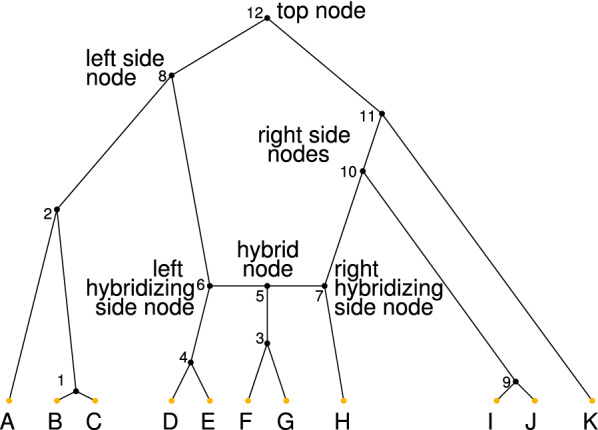
Parts of a gall. These include the top node (12), left non-hybridizing side nodes (8), right non-hybridizing side nodes (10, 11), left hybridizing side node (6), right hybridizing side node (7), and hybrid node (5). In this galled tree, leaf nodes (orange) are labeled with letters **A**–**K**. Internal nodes (black) are numbered using a postorder traversal, with child nodes assigned smaller numbers than parent nodes; at hybridization events, the subtree that receives the smallest numbers is the subtree descended from the hybrid node, and the hybrid node receives a smaller number than the hybridizing side nodes

By convention, we refer to a galled tree as a tree, even though in a technical sense, a galled tree with one or more galls is not a tree. In the literature on phylogenetic networks, a *galled network* is distinct from a galled tree, so that this term is not available for galled trees. As all trees and networks that we consider are rooted and binary, we usually omit these terms, understanding that they are implied.

It is convenient to name various features associated with a gall in a galled tree (Fig. 2). First, all nodes that are not leaf nodes are *internal nodes*, including the root. The internal nodes include the tree nodes and the reticulation nodes. For a gall with reticulation node $a_r$, ancestor node *r*, and cycle $C_r$, because we draw galled trees with the ancestors at the top of the diagram, with descent proceeding from top to bottom, we refer to the ancestor node *r* as the *top node*. The reticulation node $a_r$ is termed a *hybrid node*. All nodes in the set $C_r$ of nodes in the gall, other than the top node and the hybrid node, are *side nodes*. Each gall has two side nodes that are special; these side nodes are the nodes that are the immediate parents of the hybrid node; we call them *hybridizing side nodes* or simply *hybridizing nodes*. For visual clarity, we draw the bottom of a gall as a horizontal line, representing the idea that the hybridizing nodes instantaneously hybridize to produce the hybrid node. On this horizontal line, we always place the hybrid node between the two hybridizing side nodes.

All side nodes other than the two hybridizing side nodes are termed *non-hybridizing side nodes*. Each side node is a *left side node* or *right side node*. We use the terms “left” and “right” for convenience, associating “left” side nodes with the left side of a gall in drawings of galled trees and “right” side nodes with the right side; however, we regard a galled tree as invariant with respect to the exchange of left and right descendants of one or more top nodes. The gall has subtrees associated with each side node, the hybridizing nodes, and the hybrid node. We denote the set of subtrees associated with the gall by ${\mathcal {T}}$, the subtree descended from internal node *i* by $T_i$, and the number of internal nodes in a tree *T* by *v*(*T*).

Note that we follow Bienvenu et al. [1] in only considering networks to which it is possible to assign a chronological order of internal nodes in addition to a genealogical order. That is, supposing each node is associated with an instant in time, we disallow networks that involve such temporal impossibilities as a hybridization of node $v_1$ with a child node of $v_2$ occurring in a network that also contains a hybridization of $v_2$ with a child of $v_1$. In the same manner that Bienvenu et al. [1] consider tree-child networks and *ranked* tree-child networks, we consider galled trees and *ranked* galled trees, where a ranked galled tree is a galled tree together with its labeled history: the chronological order in which its branching—or coalescence—and hybridization events take place.

Given a set of labeled leaves of a phylogenetic tree or network, a *labeled topology* is the structure that describes the topological relationship ancestral to the leaves. The labeled topology includes both coalescences and hybridization events. Thus, for example, the labeled topology of the galled tree in Fig. 2 is obtained by disregarding the temporal sequence of the internal nodes, so that only the connectivity of the nodes is considered.

We interpret galled trees with a sense of time proceeding from the root to the leaves, all of which are contemporaneous. With this interpretation, a labeled topology might permit several distinct orders in which its coalescence and hybridization events can occur. For a given labeled topology, a *labeled history* is a specific order of its coalescences and hybridizations. That is, a labeled history of a tree or network is the labeled topology of the tree or network together with the associated temporal sequence of its internal nodes. For the example in Fig. 2, forward in time, the labeled history places the internal nodes in the sequence 12, 8, 11, 10, 2, (5, 6, 7), 3, 4, 9, 1, where 5, 6, and 7 are contemporaneous. More generally, for our enumeration of labeled histories compatible with the labeled topology of a galled tree, we treat each hybrid node as contemporaneous with its two parental nodes.

Formally, consider a galled tree labeled topology with a node set *V* including *n* leaves, an edge set *E*, and a partial order $\lesssim$ that describes ancestor–descendant relationships. In particular, two nodes $v_1,v_2$ in *V* satisfy $v_1 \lesssim v_2$ if $v_1$ lies on a path from the root node to $v_2$; a pair of edges $e_1,e_2$ in *E* can also satisfy $e_1 \lesssim e_2$ if $e_1$ lies on a path from the root node to $e_2$, and a node *v* and edge *e* can also satisfy $v \lesssim e$ if *v* is ancestral to *e*, or $e \lesssim v$ if *e* is ancestral to *v*. Trivially, a node or edge is ancestral to itself and descended from itself. Associate with each node *v* a *time* *t*(*v*), such that $t(v_1) \le t(v_2)$ if $v_1 \lesssim v_2$. For $v_1 \ne v_2$, we require $t(v_1)=t(v_2)$ if $\{v_1,v_2\}$ contains (i) a hybrid node and one of its parental hybridizing nodes; (ii) the two hybridizing nodes that are the parents of the same hybrid node; or (iii) two leaves. Otherwise, $t(v_1) \ne t(v_2)$. A labeled history is a sequence of sets of nodes $W_1, W_2, \ldots , W_n$ such that (i) for all *i* and all nodes $v_{i1}, v_{i2} \in W_i$, $t(v_{i1})=t(v_{i2})$, and (ii) for all *i*, *j* with $i<j$ and all nodes $v_i,v_j$ with $v_i \in W_i$ and $v_j \in W_j$, $t(v_i) < t(v_j)$. $W_1$ contains only the root node, and $W_n$ contains the leaves. Note that the number of sequences of sets $W_i$—the number of distinct points in time occupied by the nodes of a galled tree—is equal to the number of leaves in the galled tree and does not depend on the number of galls.

In the same way that the term “galled tree” abuses the term *tree*, we also abuse the term *subtree* by allowing a “subtree” to contain galls. Technically, a “subtree” that contains galls is not a tree, but it is convenient to think of it as tree-like. Hence, each internal node of a galled tree has a subtree to which it is ancestral; for a hybridizing node that has two child nodes, one of which is a hybrid node, that hybridizing node is immediately ancestral to the root of a subtree that includes the child node that is not the hybrid node. When referring to subtrees “descended” from an internal node, we are describing the subtrees rooted at children of the node. A non-hybridizing side node has exactly one such subtree, rooted at one of its child nodes; the other child node is part of its associated gall.

### Labeled histories for trees

We recall results concerning the enumeration of labeled histories for trees (without galls). The number of labeled histories for a rooted binary tree with *n* leaves has been obtained both recursively and nonrecursively. We will have occasion to use both the recursive and nonrecursive formulas, as our enumeration of galled trees follows the reasoning of the recursive approach, and the nonrecursive formula is convenient in steps that count labeled histories for non-galled subtrees of a galled tree.

The root of a binary tree has two subtrees. To obtain a labeled history for a full labeled binary tree, the internal nodes of the two subtrees can be arranged in any order in relation to one another, maintaining the order within each subtree. The number of labeled histories of a tree is the product of the numbers of labeled histories of the two subtrees and the number of ways in which the internal nodes of the two subtrees can be interwoven once the subtree labeled histories are fixed. Hence, the recursive formula for the number of labeled histories of a tree *T* whose subtrees $T_{\ell }$ and $T_r$ have $v(T_{\ell })$ and $v(T_r)$ internal nodes, respectively, is

1 $$\begin{aligned} L_H(T) = L_H(T_{\ell }) \, L_H(T_r) \, { {v(T_{\ell }) + v(T_r) \atopwithdelims ()v(T_r)}}, \end{aligned}$$

where $L_H(T)=1$ if *T* has a single leaf or if *T* is a 2-leaf tree [3, 11].

In nonrecursive form (Lemma 1 of [25]), the number of labeled histories is

2 $$\begin{aligned} L_H(T) = \frac{(n-1)!}{\prod _{i \in V^0(T)} v(T_i)}, \end{aligned}$$

where $V^0(T)$ is the set of internal nodes of *T* (including the root) and $T_i$ is the subtree descended from internal node *i*.

## Example

To count labeled histories of galled trees, we use a recursive approach that generalizes the recursive count for labeled histories of a tree without galls. Informally, we can view a galled tree as a network that is structurally similar to a true tree. In particular, in a gall, side nodes and the hybrid node each give rise to descendant subtrees, which might themselves include galls. Note that if such a subtree includes galls, then it is more accurately termed a subnetwork; for convenience, we continue to call it a subtree.

We begin from the root node of the galled tree. If the root is not the top node of a gall, then we proceed toward its child nodes as in the recursive enumeration of labeled histories for trees. We count labeled histories for the left subtree and for the right subtree, and we count ways that these labeled histories can be interwoven in relation to one another.

If, instead, the root node is a top node of a gall, then we introduce a new recursive function that enumerates labeled histories for the subtrees of all side nodes of the gall, both hybridizing nodes of the gall, and the unique hybrid node of the gall, and that enumerates the ways in which the labeled histories of these subtrees can be interwoven.

We apply this recursive function proceeding down through the galled tree. Each gall contains, at minimum, three associated subtrees—two descended from the two hybridizing side nodes, and one from the hybrid node. Hence, the recursive component of our enumeration of labeled histories associated with a galled tree considers at least three subtrees; in other words, it proceeds by noting that all galls are divided into three or more parts.

**Fig. 3:**
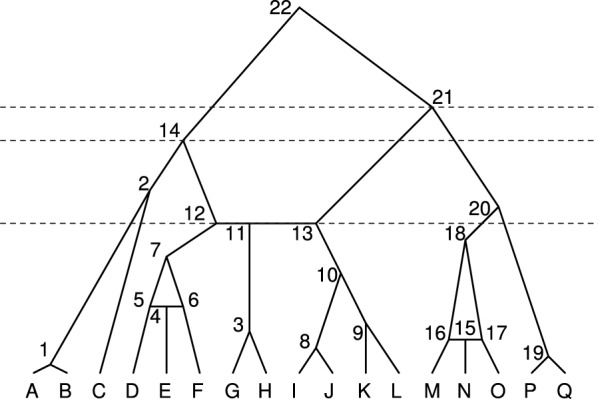
An example galled tree. This tree has three galls. One gall has a top node at the root (node 22), with two side nodes on the left and two on the right. A second gall is in the subtree $T_L$ descended from the left hybridizing side node of the first gall (node 12); this second gall has only one side node on the left and one side node on the right (its hybridizing side nodes). A third gall is descended from the right non-hybridizing side node of the first gall (node 21)

Figure 3 shows an example of a small galled tree. Considering the gall at the root node (node 22), subtrees $T_{\ell 1}$ (descended from node 14, a left non-hybridizing side node), $T_L$ (descended from node 12, the left hybridizing side node), $T_C$ (descended from node 11, the hybrid node), $T_R$ (descended from node 13, the right hybridizing side node), and $T_{r1}$ (descended from node 21, a right non-hybridizing side node) are galled trees with 1, 1, 1, 2, and 3 labeled histories, respectively. For $T_{\ell 1}$, $T_C$, and $T_R$, the subtrees are trees in the usual sense, and the numbers of labeled histories follow eqs. 1 and 2. Galled subtree $T_L$ trivially possesses only one labeled history. For $T_{r1}$, both its subtrees each trivially possess a single labeled history. The left subtree possesses a coalescence at node 18 and a hybridization represented by simultaneous nodes 15–17, and the right subtree has the one coalescence at node 19. The right subtree can be arranged in one of three ways in relation to the left subtree: node 19 nearer in time to the root than node 18, between node 18 and nodes 15–17, or more recent than nodes 15–17. Hence, the number of labeled histories for $T_{r1}$ is 3.

For the gall at the root node, the left non-hybridizing side node can be arranged in relation to the right non-hybridizing side node. The number of arrangements of these non-hybridizing side nodes in relation to one another is ${{1+1} \atopwithdelims (){1}} = 2$, as we are counting arrangements of 1 left non-hybridizing side node and 1 right non-hybridizing side node. In general, when including the hybridizing side nodes in node counts $n_{\ell }$ and $n_{r}$, the number of arrangements of $n_{\ell }$ left side nodes and $n_{r}$ right side nodes is ${n_{\ell } + n_{r}-2} \atopwithdelims (){n_{\ell } -1}$.

The arrangement of the side nodes creates “time periods.” The arrangement depicted in the example has three nontrivial time periods. The first lies below the first side node (node 21), the next is below the next side node (node 14), and a final period lies after the hybridization (nodes 11–13). The internal nodes in the subtree of a side node can only be placed in the periods subsequent to the side node itself, so the number of time periods available for such a subtree is determined by the arrangement of the side nodes on the gall. The internal nodes in the subtrees of the side nodes are then distributed into the available time periods. For each time period, the number of ways of arranging all nodes assigned to that time period across the various subtrees is given by a multinomial coefficient. The number of labeled histories for each assignment of internal nodes to time periods is then the product across time periods of the associated multinomial coefficients for the time periods. The total number of labeled histories for each ordering of the side nodes on the gall is the sum across assignments of internal nodes to time periods of the number of labeled histories for each assignment.

In the example, we have two arrangements for the side nodes: (14, 21) and (21, 14). We first consider arrangement (21, 14) depicted in Fig. 3. We calculate the number of ways to distribute the nodes from the subtrees into the time periods, and for each distribution we then calculate the numbers of arrangements within each of the time periods. There are three time periods: (i) between 21 and 14, (ii) between 14 and the hybridizing side nodes, and (iii) below the hybridizing side nodes. The ordering of the internal nodes within a subtree does not affect the permissible placements of these nodes within the time periods. Therefore, the number of labeled histories for a particular ordering of the side nodes on the gall is the product of two quantities: (1) the product across subtrees of the numbers of labeled histories for the subtrees, $\prod _{T_i}L_H(T_i)$, and (2) the number of ways that these labeled histories can be interwoven in relation to one another for the fixed ordering of the side nodes on the gall.

To count this latter quantity, we must consider all possible placements of subtree nodes into time periods. We define an “event” to be either a (non-hybridizing) coalescence or a hybridization. This concept of an event corresponds to that of an internal node used in the recursive calculation of the number of labeled histories for trees in eq. 1. Each non-hybridizing tree node corresponds to a coalescence event. We denote the number of events in a subtree $T_i$ as $v(T_i)$. The total number of events in any galled tree is two times the number of hybrid nodes subtracted from the total number of internal nodes, because each hybridization event is represented by three simultaneous internal nodes—the hybrid node and two hybridizing nodes. For a subtree $T_i$, the number of time periods in which the events of that subtree can occur, $p(T_i)$, depends on the arrangement of the side nodes. The events in the subtree cannot occur in time periods preceding the side node from which the subtree descends. For each subtree $T_i$, we count the available time periods for the events of $T_i$. When the left side node, node 14, occurs after the right side node, node 21, the numbers of time periods available to $T_{\ell 1}$, $T_L$, $T_C$, $T_R$, and $T_{r1}$ are 2, 1, 1, 1, and 3, respectively.

The number of ways to distribute *n* events—which have already been ordered—into *t* time periods is ${n+t-1} \atopwithdelims ()n$. Let $v(T_i)$ denote the number of events in subtree $T_i$. When node 21 precedes node 14, the total number of ways to arrange the subtree events into time periods is $\prod _{T_i}{{v(T_i) + p(T_i) - 1} \atopwithdelims (){v(T_i)}}$, or in this case, ${{2+2-1}\atopwithdelims (){2}} {{2+1-1}\atopwithdelims (){2}} {{1+1-1}\atopwithdelims (){1}} {{3+1-1}\atopwithdelims (){3}} {{4+3-1}\atopwithdelims (){4}} = 45$.

Consider one of these 45 arrangements of events into time periods, say, in which tree $T_{\ell 1}$ has its nodes 1 and 2 in the third and second time periods, respectively, and $T_{r1}$ has its internal node 20 in the second period and nodes 15–19 in the third (Fig. 3). For $T_L$, $T_C$, and $T_R$, the internal nodes are trivially in the final (third) period. For each of the periods, we must count the number of orderings permitted for internal nodes allowed within the period. The first period (between nodes 21 and 14) has no nodes, so this period trivially has one arrangement. Nodes 2 and 20 occur in the second period between node 14 and the hybridization represented by nodes 11-13. Because nodes 2 and 20 are from different subtrees, they can be arranged in either of two orders in relation to one another, so there are 2 possible orderings within the second time period. The final period has 10 events; 1, 2, 1, 3, and 3 from $T_{\ell 1}$, $T_L$, $T_C$, $T_R$, and $T_{r1}$, respectively. Hence, there are ${10 \atopwithdelims (){1,2,1,3,3}} = 50,400$ possible orderings of events in that period. For the fixed subtree labeled histories and distribution of events across periods shown in Fig. 3, the number of labeled histories is $2 \times 50,400 = 100,800$.

We repeat this procedure for each of the 45 cases, for each case counting its associated product of multinomial coefficients. We will see that by careful indexing, the appropriate product of multinomial coefficients can be obtained generally. Summing across the 45 cases, we obtain 2,162,160 labeled histories.

Keeping the labeled histories of the subtrees fixed, we count labeled histories for the other arrangement of the side nodes with node 14 preceding node 21, obtaining 1,801,800; this calculation sums across ${{2 + 3 -1 }\atopwithdelims (){2}} {{2 + 1 -1 }\atopwithdelims (){2}} {{1 + 1 -1 }\atopwithdelims (){1}} {{3 + 1 -1 }\atopwithdelims (){3}} {{4 + 2 -1 }\atopwithdelims (){4}} = 30$ cases. Multiplying by $\prod _{T_i}L_H(T_i) = 2 \times 1 \times 1 \times 1 \times 3 = 6$, the product across subtrees of the numbers of labeled histories for the subtrees, the total number of labeled histories is $6 \times (2,162,160 + 1,801,800) = 23,783,760$.

We are now ready for the general computation.

## General algorithm

Our general result follows the example to recursively calculate the number of labeled histories in any galled tree. Consider a galled tree *T* with root node *v*. Either *v* is the top node of a gall or it is not the top node of a gall.

If *v* is *not* a top node, then the number of labeled histories of the tree rooted at *v* is the product of the numbers of labeled histories for the two subtrees of *v* and the number of ways in which those subtrees can be interwoven. We recursively proceed to the children of *v* to count labeled histories for the subtrees descended from these children.

If *v* is the top node of a gall, then we proceed as in the example. Denote by *G* the gall for which *v* is the top node. Suppose *G* has left non-hybridizing side nodes $g_{\ell 1}, g_{\ell 2}, \ldots , g_{\ell N}$ and right non-hybridizing side nodes $g_{r1}, g_{r2}, \ldots , g_{rM}$. The subtrees of the galled tree are then $T_{L}$ from the left hybridizing side node, $T_R$ from the right hybridizing side node, $T_C$ from the hybrid node, and $T_{\ell 1}, T_{\ell 2}, \ldots , T_{\ell N}, T_{r1},T_{r2}, \ldots , T_{rM}$ from the non-hybridizing side nodes. We can count the number of labeled histories for the subtree defined by the gall rooted at *v*, supposing the numbers of labeled histories are known for all these various subtrees.

1. We enumerate the possible orderings of the left side nodes in relation to the right side nodes. Let the set of all orderings of side nodes of the gall rooted at *v* be $S_v$. The ordering of the left side nodes is fixed and the ordering of the right side nodes is fixed; this step counts the ways in which the left and right side nodes can be interwoven. Hence, $S_v$ has cardinality ${N + M \atopwithdelims ()N}$. Each arrangement of the side nodes defines “time periods” between side nodes, into which other nodes can be placed.
2. We separately consider each of the ${N + M \atopwithdelims ()N}$ arrangements of the non-hybridizing side nodes—the elements of $S_v$—and enumerate assignments of internal nodes of the subtrees descended from the non-hybridizing side nodes (and the hybridizing nodes and hybrid node) into time periods. Let the set of all assignments in an arrangement of side nodes $s \in S_v$ be *X*(*s*). The number of assignments of these internal nodes depends on the numbers of time periods that are available to the various subtrees. Internal nodes in a subtree can only be assigned into time periods that occur after the non-hybridizing side node (or hybridizing node or hybrid node) from which the subtree descends. Let the number of time periods available to subtree $T_i$ be $p(T_i)$. Recalling that $v(T_i)$ is the number of coalescence or hybridization events in the galled tree $T_i$, the number of ways to arrange the internal nodes of subtree $T_i$ into subtrees is ${{v(T_i) + p(T_i) - 1} \atopwithdelims ()v(T_i)}$. That is, because each subtree can have its nodes assigned without considering the assignment of other subtrees, the total number of assignments of internal nodes to time periods is the product of the number of assignments over all subtrees. Therefore *X*(*s*) has cardinality $\prod _i {{v(T_i) + p(T_i) - 1} \atopwithdelims ()v(T_i)}$, where the product traverses $N+M+3$ nodes: the $N+M$ non-hybridizing side nodes as well as the two hybridizing nodes and the hybrid node.
3. For each assignment of internal nodes to time periods, we count the number of orderings of those internal nodes within the time periods. For each assignment, we list the numbers of nodes assigned to each of the $N+M+1$ time periods. We construct a matrix for assignment *x*, $A_x \in {\mathbb {Z}}^{(N + M + 3) \times (N + M +1)}$, where entry $A_{x(i,j)}$ is the number of events from subtree *i* that are placed in time period *j* and $\sum _{j=1}^{N+M+1} A_{x(i,j)} = v(T_i)$. The number of labeled histories for the specific assignment is then equal to
  $$\begin{aligned} \prod _{j=1}^{N+M+1} { \sum _{i=1}^{N+M+3} A_{x(i,j)} \atopwithdelims ()A_{x(1,j)}, A_{x(2,j)}, \ldots , A_{x(N+M+3,j)} }. \end{aligned}$$
4. We combine steps 1–3 to obtain the number of labeled histories for the gall whose top node is *v*. In particular, we now have the total number of labeled histories for each specific arrangement of the side nodes and fixed set of labeled histories for the subtrees. The number of labeled histories for the subtree defined by the gall is then the sum of the number of labeled histories across each arrangement of the side nodes on the gall multiplied by the numbers of labeled histories of the subtrees. In other words, the number of labeled histories for the gall rooted at *v* is
  3 $$\begin{aligned} & \bigg (\prod _{k\in {\mathcal {T}}} L_H(T_k) \bigg ) \\ & \quad \times \sum _{s \in S_v} \sum _{x \in X(s)} \prod _{j=1}^{N+M+1} { \sum _{i=1}^{N+M+3} A_{x(i,j)} \atopwithdelims ()A_{x(1,j)}, A_{x(2,j)}, \ldots A_{x(N+M+3,j)} }, \end{aligned}$$
  where $${\mathcal {T}} = \{ T_{\ell 1}, T_{\ell 2}, \ldots , T_{\ell N}, T_{r1},T_{r2}, \ldots , T_{rM}, T_L, T_R, T_C\}$$.

We recursively enumerate the labeled histories of a galled tree, applying the steps beginning from the tree root and proceeding to the leaves through each top node of a gall.

## Small galled trees

We exhaustively count the labeled histories for all galled trees with six or fewer leaves. For each unlabeled galled tree with six or fewer leaves, Tables 1, 2, 3, 4, and 5 report the numbers of labeled histories associated with an arbitrary labeling of the galled tree; a summary appears in Table 6.

### Enumeration of small galled trees

First, we enumerate all unlabeled galled trees with six or fewer leaves. This enumeration proceeds by first listing all trees with no galls. The number of such trees follows the Wedderburn-Etherington numbers,

$$\begin{aligned} U_1= & {} 1 \\ U_n= & {} \bigg (\sum _{k=1}^{\frac{n}{2}-1} U_k U_{n-k} \bigg ) + \frac{ U_{\frac{n}{2}} (U_{\frac{n}{2}} + 1 )}{2}, \text { even } n\ge 2 \\ U_n= & {} \sum _{k=1}^{\frac{n-1}{2}} U_k U_{n-k}, \text { odd } n\ge 3. \end{aligned}$$

The number of unlabeled trees with *n* leaves is obtained by combining all possible pairs of subtrees, one with *k* leaves, $1 \le k \le \lfloor \frac{n}{2} \rfloor$, and the other with $n-k$ leaves.

To enumerate all galled trees with *n* leaves, we follow a similar procedure of combining smaller galled trees to form a galled tree of a fixed number of leaves. We consider two cases: either the root node is the top node of a gall or it is not. If the root node is not the top node of a gall, then we recursively form galled trees in the same way as in the case of no galls, by combining pairs of smaller galled trees.

For the other case, if the root node is a top node of a gall, then we consider all galls that are possible at the top of the tree. For a galled tree with *n* leaves, a gall has a minimum of 3 subtrees: two for the hybridizing nodes and one for the hybrid node. The maximum number of subtrees emanating from the gall is *n*, corresponding to the case in which there are $n-3$ non-hybridizing side nodes, each of which has a leaf node for its associated subtree.

The non-hybridizing side nodes can be placed into the left and right sides of the gall in each of multiple ways. Without loss of generality, suppose that the number of non-hybridizing side nodes on the left side is always greater than or equal to the number on the right, $n_{\ell } \ge n_{r}$. The number of subtrees emanating from the gall then equals $n_{\ell } + n_{r} + 3$ (here we exclude the hybridizing side nodes from $n_{\ell }$ and $n_{r}$). We enumerate the ways to partition *n* leaves into $n_{\ell } + n_{r} + 3$ labeled categories—the number of compositions of *n* into $n_{\ell } + n_{r} + 3$ parts, where each part represents a specific one of the subtrees. Each subtree of each composition is a smaller galled tree.

For $n_{\ell } \ne n_{r}$, we proceed by allowing each combination of smaller galled trees of the $n_{\ell } + n_{r} + 3$ sizes specified. In the case with $n_{\ell }=n_{r}$, we must be careful not to double-count. Write a vector *c* representing the composition that counts leaves in the $n_{\ell } + n_{r} + 3$ subtrees. The composition is ordered from “left” to “right,” starting from the most ancestral left side node, proceeding from ancestor to descendant to the left hybridizing side node, then the hybrid node and the right hybridizing side node, and proceeding from descendant to ancestor to the most ancestral right side node.

A composition can be “palindromic” in the sense that it is invariant with respect to inversion of the order of its terms; for example (3, 2, 4, 2, 3) is a palindromic composition of 14, whereas (2, 2, 3, 3, 4) is non-palindromic. For a non-palindromic composition *c*, let $c'$ be the composition obtained by inverting the order of its terms: (4, 3, 3, 2, 2) for (2, 2, 3, 3, 4), for example. We consider two cases:

(i) For each pair of non-palindromic compositions of $n_{\ell } + n_{r} + 3$, $(c,c')$, we only consider one of the two.
(ii) For each palindromic composition *c*, we enumerate the set of all possible lists of $n_{\ell } + 1$ subtrees for the left side nodes, including the left hybridizing side node, in some specified order. We choose two lists in this set, allowing replacement, one for the subtrees of the left side nodes, and one for the subtrees of the right side nodes (proceeding backward from the end of the composition). If the two lists are different, then we always use for the left side nodes the list that appears earlier in the order. To complete the enumeration, we combine all possible lists of subtrees for the left and right side nodes with all possible subtrees for the hybrid node.

Note that for a tree with *n* leaves, the maximal number of galls is $\lfloor \frac{n-1}{2} \rfloor$. To verify this claim, start with a galled tree with a single gall and three leaves—the minimum number of leaves for a galled tree, as the hybridizing and hybrid nodes must each have at least one descendant. Each subsequent gall adds at least two leaves, as a gall can replace at most one existing leaf. Therefore, the minimum number of leaves for a galled tree with *g* galls is $2g+1$, so that $n \ge 2g + 1$, or $g \le \lfloor \frac{n-1}{2} \rfloor$.

### Labeled histories for small galled trees

Examining Tables 1, 2, 3, 4, and 5, we can observe the pattern that for a fixed number of leaves, for trees with no galls, the number of labeled histories increases with increasing tree balance. Caterpillar trees, in which there exists an internal node descended from all other internal nodes, possess only one labeled history.

**Fig. 4:**
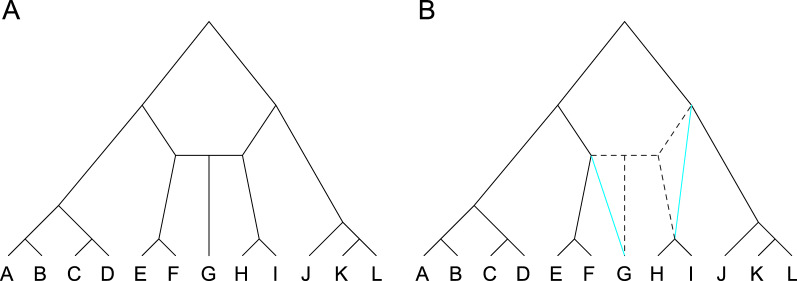
Removing a gall does not decrease the number of labeled histories. **A** A tree with one gall. **B** A tree obtained from **A** via a transformation that removes the gall; we remove the hybrid node and one of the hybridizing side nodes, choosing the right hybridizing side node arbitrarily here. We then add two edges, between the left hybridizing side node and the child of the hybrid node, and between the parent and remaining child of the right hybridizing side node (blue). Each labeled history for **A** has a corresponding labeled history in **B**

In general, trees with more galls tend to have fewer labeled histories than trees with fewer galls. Indeed, we can always remove a gall while retaining the same number of leaves and retaining or increasing the number of labeled histories. Consider a galled tree *T*. We delete the hybrid node and the right hybridizing side node. We then add an edge that joins the left hybridizing side node and the child of the hybrid node, and another edge that joins the parent and child of the right hybridizing side node (Fig. 4). The resulting galled tree $T'$ has the same number of leaves as *T*. Further, each labeled history for *T* continues to have an associated labeled history for $T'$—the coalescence of the left hybridizing side node, hybrid node, and right hybridizing side node in *T* is now indexed only by the left hybridizing side node in $T'$. Hence $T'$ has at least as many labeled histories as *T*, and indeed might have more, as the nodes in the subtree of the former right hybridizing side node of *T* can now move above the former left hybridizing node, and hence are less constrained in $T'$.

As a corollary of this argument, given a fixed number of leaves, in the set of galled trees that possess the largest number of labeled histories—a set that contains at least one and potentially more than one element—at least one element is a tree with no galls. Other consequences include: (i) for galled trees with *n* leaves, the galled tree that maximizes the number of labeled histories among galled trees with *g* galls, $g \ge 1$, has no more labeled histories than the galled tree that maximizes the number of labeled histories among galled trees with $g-1$ galls. Further, (ii) no galled tree with *n* leaves has more labeled histories than the labeled topology (with no galls) that maximizes the number of labeled histories.

## Discussion

We have devised a method for enumerating the labeled histories for rooted binary labeled galled trees. The method generalizes the classic enumeration of labeled histories for labeled topologies, extending it to a simple family of phylogenetic networks. We have applied our new algorithm to enumerate labeled histories both in an illustrative example and exhaustively for small galled trees with at most six leaves. In this latter analysis, we have found that for a fixed number of leaves, adding galls generally reduces the number of labeled histories.

Labeled histories have long been a focus of studies in phylogenetics [6, 11], appearing often in calculations that describe the probability that random trees produce specified shapes under evolutionary models [18, 24, 26]. Recent studies have been expanding the sense in which labeled histories are considered. For example, King & Rosenberg [14] examined a concept of labeled histories in which simultaneous binary mergers of lineages are permitted. Bienvenu et al. [1] explored the possibility of providing labeled histories to phylogenetic networks, specifically tree–child networks. They emphasized that ranked tree-child networks, which impose a temporal structure on tree-child networks, have biological relevance because chronological processes are, by definition, rankable. Bienvenu et al. [1] suggested the problem of enumerating labeled histories for a tree–child network, noting the difficulty that networks do not possess the recursive properties of trees. We have found that for the galled trees, a subset of the tree–child networks, we can continue to use a tree-like recursive approach to enumerate labeled histories. To our knowledge, our calculation provides the first enumeration of labeled histories beyond trees to a class of phylogenetic networks.

Our enumeration facilitates the understanding of factors that affect the number of labeled histories for galled trees. We found that for a fixed number of leaves, increasing the number of galls does not increase, and often decreases, the number of labeled histories. We have only considered small numbers of leaves, and as the number of leaves increases, it will be of interest to explore the effect on the number of labeled histories of gall locations—for example, with a top node located or not located at the tree root, or with multiple galls descended from one another or not descended from one another. For labeled topologies with a fixed number of leaves, the topology with the maximum number of labeled histories has a high level of “balance” [10], and permitting galls does not change the identity of the galled tree with the maximal number of labeled histories. Future work, however, can examine more generally the effect of balance on the number of labeled histories for galled trees.

An important aspect of our analysis is that the sense in which we consider galled trees has an explicit temporal ordering, in which each gall possesses two hybridizing nodes and a hybrid node that are contemporaneous. With their explicit potential to be temporally ordered, the rooted galled trees here and galled trees in other studies are not generally precisely identical, as the temporal requirement we have imposed is a case of the recent approach of Bienvenu et al. [1] and has not yet been frequently assumed. We have provided an enumeration algorithm for the galled trees we consider; the counts of 1, 1, 2, 6, 20, and 72 for the numbers of rooted unlabeled galled trees for 1 to 6 leaves (Table 6) differ from counts and formulas reported in related enumerative studies [2, 4, 7, 8, 21]. In studies of labelings for galled trees and phylogenetic networks more generally, care is needed in recognizing the precise set of objects under consideration.

The enumeration of labeled histories is more computationally challenging for galled trees than for trees with no galls. Whereas the evaluation of the number of labeled histories for trees with no galls can use a simple nonrecursive formula (eq. 2), the algorithmic enumeration of labeled histories for galled trees requires a number of steps that, for some trees, increases at least exponentially in the number of leaves. In enumerating labeled histories, the first step for each gall is to sum over all orderings of the left side nodes in relation to the right side nodes. Consider a family of trees $T_n$ with $n=4k+1$ leaves for $k \ge 1$. Suppose $T_n$ has a root gall with *k* left side nodes and *k* right side nodes, each with two descendant leaves, for a total of 4*k* leaves descended from the side nodes; the last leaf is descended from the hybrid node. The number of orderings of left and right side nodes over which we must sum in eq. 3 is ${2k \atopwithdelims ()k}$, a quantity that increases exponentially, as $4^k/\sqrt{\pi k}$, or $(\sqrt{2})^n \sqrt{2/[\pi (n-1)]}$. Considering galled trees more generally, computation time increases with the number of side nodes in galls, the number of leaves descended from those side nodes, and the number of galls descended from one another along a path from the root to the leaves. For a fixed number of leaves *n*, maximizing any of these three quantities occurs by reducing the other two, so that the configuration of galls and leaves that maximizes computation time for the enumeration—as well as the complexity of the computation itself—remains unknown.

The potential to embed galled trees in a recursive framework is central to our solution for enumerating labeled histories for labeled galled trees. The solution enables us to treat galls similarly to internal nodes in standard phylogenetic trees by defining subtrees that descend from nodes of a gall. It is possible that a more general solution to the enumeration of labeled histories for other classes of phylogenetic networks could rely on creatively finding such recursive properties.

**Table 1:** Number of labeled histories for galled trees with at most 4 leaves

Number of leaves	Number of galls	Galled tree	Number of labeled histories
1	0		1
2	0		1
3	0		1
3	1		1
4	0		1
4	1		1
4	0		2
4	1		1
4	1		1
4	1		1

Galled trees with different numbers of galls appear in different colors (0, black; 1, orange; 2, purple). For each unlabeled galled tree shown, an arbitrary labeling of the leaves is assumed, and the number of labeled histories associated with that arbitrary labeling is shown

**Table 2:** Number of labeled histories for galled trees with 5 leaves

Number of leaves	Number of galls	Galled tree	Number of labeled histories
5	0		1
5	1		1
5	0		2
5	1		1
5	1		1
5	1		1
5	0		3
5	1		3
5	1		1
5	2		1
5	1		2
5	1		2
5	1		1
5	2		1
5	1		2
5	1		1
5	1		1
5	1		1
5	1		1
5	1		1

**Table 3:** Number of labeled histories for galled trees with 6 leaves (first part)

Number of leaves	Number of galls	Galled tree	Number of labeled histories
6	0		1
6	1		1
6	0		2
6	1		1
6	1		1
6	1		1
6	0		3
6	1		3
6	1		1
6	2		1
6	1		2
6	1		2
6	1		1
6	2		1
6	1		2
6	1		1
6	1		1
6	1		1
6	1		1
6	1		1

**Table 4:** Number of labeled histories for galled trees with 6 leaves (second part)

Number of leaves	Number of galls	Galled tree	Number of labeled histories
6	0		4
6	1		4
6	0		8
6	1		4
6	1		4
6	1		4
6	0		6
6	1		6
6	2		6
6	1		1
6	2		1
6	1		2
6	2		1
6	2		1
6	2		1
6	1		3
6	2		3
6	1		3
6	2		3
6	1		3
6	2		3
6	1		6
6	1		1
6	2		1

**Table 5:** Number of labeled histories for galled trees with 6 leaves (third part)

Number of leaves	Number of galls	Galled tree	Number of labeled histories
6	1		2
6	2		1
6	2		1
6	2		1
6	1		3
6	2		3
6	1		3
6	1		3
6	1		3
6	1		1
6	2		1
6	1		2
6	1		2
6	1		2
6	2		2
6	1		2
6	1		2
6	2		2
6	1		2
6	1		2
6	1		2
6	1		2
6	1		2
6	1		2
6	1		2
6	1		2
6	1		2
6	1		2

**Table 6:** Features of galled trees with 6 or fewer leaves

Number of leaves	Number of galled trees	Number of galled trees with *g* galls			Maximum number of labeled histories among galled trees with *g* galls		
		$g=0$	$g=1$	$g=2$	$g=0$	$g=1$	$g=2$
1	1	1	0	0	1	–	–
2	1	1	0	0	1	–	–
3	2	1	1	0	1	1	–
4	6	2	4	0	2	1	–
5	20	3	15	2	3	3	1
6	72	6	48	18	8	6	6

The features are extracted from Tables 1, 2, 3, 4, and 5

## Data Availability

Not applicable.
